# The genomic characterization of three *Microbacterium foliorum*-specific bacteriophages, “Nucci,” “MCubed,” and “QMacho”

**DOI:** 10.1128/mra.00203-24

**Published:** 2024-04-10

**Authors:** Jennifer Cook Easterwood, Joanna Mantis Katsanos, Jenna Lloyd

**Affiliations:** 1 Department of Biology, Queens University of Charlotte, Charlotte, North Carolina, USA; Department of Biology, Queens College, Queens, New York, USA

**Keywords:** genome, bacteriophage genetics

## Abstract

Nucci, MCubed, and QMacho are microbacteriophages that were isolated from soil samples in Charlotte, NC. They were classified into EA10, EA2, and EB clusters, respectively. Nucci and MCubed each had 63 predicted genes, while QMacho had 73 predicted genes.

## ANNOUNCEMENT

We report the genome sequences of three microbacteriophages (Nucci, MCubed, and QMacho) isolated using the host *Microbacterium foliorum* NRRL B-24224 from soil samples collected in Charlotte, NC (Table 1). These microbacteriophages represent clusters EA10, EA2, and EB, which contribute to the greater understanding of the diversity and abundance of microbacteriophage species in the Southeast. This information can provide a better understanding of their relationship to the local soil ecosystem (1).

**TABLE 1:** Bacteriophage names, information, and genomic characteristics

Phage name (GenBank accession number)	Soil sample collection site	Isolation year	Plaque morphology	Plaque size (mm)	Capsid size (nm)	Tail length (nm)	Approximate shotgun coverage (fold)	Genome length (bp)	Genome end characteristic	G-C content (%)	No. of open reading frames	No. of tRNAs	Cluster
Nucci (MN096367)	35.16569 N, 80.759107 W	2018	Clear, small	1	60	121	2,373	40,373	Circularly permuted	63.7	63	0	EA10
MCubed (MN096378)	35.16569 N, 80.759107 W	2018	Hazy, small	1	51	140	235	40,381	Circularly permuted	62	63	0	EA2
QMacho (OP434443)	35.189512 N, 80.831178 W	2021	Clear, medium sized	2	61	136	548	41,685	3′ sticky overhang	67	73	3	EB

**Fig 1:**
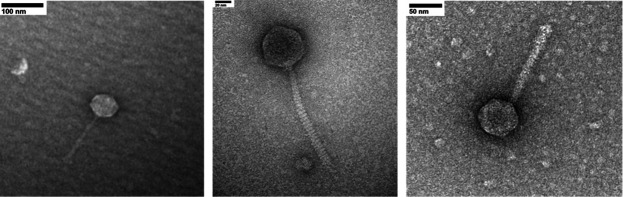
Transmission electron microscopy revealed that Nucci, MCubed, and QMacho (left to right) belong to the Siphoviridae viral family. Negative staining was performed using 2% uranyl acetate, and specimens were examined at an accelerating voltage of 120 kV using the JEM 1400Plus transmission electron microscope.

Using protocols in the SEA-PHAGES discovery guide (https://seaphagesphagediscoveryguide.helpdocsonline.com/home), we isolated the microbacteriophages using enriched isolation and the host *Microbacterium foliorum* grown in PYCa liquid media at 30°C for 2 days. Isolation was followed by three rounds of plaque assays to purify the phage, ensuring uniform plaque morphology (Table 1), followed by production of high-volume lysates to amplify the phage. Negative staining transmission electron microscopy revealed that all three have siphovirus morphology (Fig. 1). A representative virion for each phage was used to determine capsid and tail measurements using ImageJ v.1.53k.(2) (Table 1).

Genomic DNA was isolated from a high-titer lysate using the Wizard DNA Clean-Up kit (Promega) and prepared for sequencing with a NEB Ultra II DNA Kit. Genomes were sequenced with an Illumina MiSeq instrument. Nucci yielded 717,195 reads of 150-base single-end reads; MCubed yielded 334,594 reads of 150-base single-end reads, while QMacho yielded 258,988 reads of 150-base single-end reads. Raw reads were assembled using Newbler v.2.7 (3) with default settings. Consed v.29 (4) was used to check for genomic termini, accuracy, and completeness for quality control (5).

Auto-annotation of these genomes was performed using DNA Master v.5.23.6 (http://cobamide2.bio.pitt.edu/computer.htm) with protein-coding regions predicted by Glimmer v.3.02 (6) and GeneMark v.2.5p (7) using the default settings described in Howard Hughes Medical Institute’s SEA-PHAGES Bioinformatics Guide (https://seaphagesbioinformatics.helpdocsonline.com/home). Gene starts were predicted using Starterator (http://seaphages.org/software/), while protein function was determined using NCBI BLASTp v.2.7 (8), Phamerator (9), and HHPred v.3.0beta (10). Characteristics of the genomes and viral clusters are included in Table 1, and representative virion morphologies are included in Fig. 1.

Nucci contained 63 predicted protein-coding regions with 24 putative functions, MCubed contained 63 predicted protein-coding genes with 22 putative functions, and QMacho contained 73 predicted protein-coding genes with 31 putative functions. QMacho also contained three tRNA sequences predicted by Aragorn v1.1 (11) and tRNAscan-SE v.2.0.6 (12) and a putative translational frameshift in the tail assembly chaperone genes. MCubed and QMacho each had one transmembrane protein predicted by TMHMM v.2.0 (13) and SOSUI v.1.11 (14)

Clustal Omega alignment (https://www.ebi.ac.uk/Tools/msa/clustalo/) (15) using default settings indicated an 83.73% average nucleotide sequence identity (ANI) between Nucci and Mandalorian and an 83.95% ANI with Quartz. MCubed had a 96.66% ANI with Eleri and a 96.48% ANI with Sansa, all members of the EA cluster, a microbacteriophage cluster. QMacho had a 93.72% ANI with Swervy, a member of the EB cluster, also a microbacteriophage cluster.

## Data Availability

These genomes are available at GenBank under accession numbers MN096367 (Nucci), MN096378 (MCubed), and OP434443 (QMacho). Sequence Read Archive numbers are SRX20916071 (Nucci), SRX20916070 (MCubed), and SRX20916072 (QMacho).
